# Feasibility and acceptability of “LiCPain” pilot randomised controlled trial of continuous subcutaneous infusion of lidocaine or placebo for people with neuropathic cancer pain: a qualitative study of patient and carer perceptions and experiences

**DOI:** 10.1186/s12904-026-02043-x

**Published:** 2026-03-17

**Authors:** Jessica T. Lee, Meera Agar, Jane L. Phillips, Angela Rao, Emily Huang, Megan Ritchie, Belinda Fazekas, Priyanka Vandersman, Ghauri Aggarwal, Dipti Mittal, Caitlin Sheehan, Rajesh Aggarwal, Davinia Seah, Marian Kow, Rachel George, Richard Chye, Anthony Linton, Bev Noble, David Currow, Chadi Ayoub, Kat Urban, Christine Sanderson, Belinda E. Butcher, Andrew McLachlan, Melanie R. Lovell

**Affiliations:** 1 Improving Palliative, Aged and Chronic Care Through Clinical Research and Translation (IMPACCT), Faculty of Health, University of Technology Sydney, Sydney, NSW Australia; 2 Concord Centre for Palliative Care, Concord Repatriation General Hospital, Concord, NSW Australia; 3 Concord Clinical School, University of Sydney, Concord, NSW Australia; 4 HammondCare, Greenwich Palliative and Supportive Care Services, Sydney, NSW Australia; 5 Northern Clinical School, University of Sydney, St Leonards, New South Wales Australia; 6 WriteSource Medical Pty Ltd, North Sydney, New South Wales Australia; 7 Palliative Care, Calvary Healthcare, Kogarah, NSW Australia; 8 Sydney Pharmacy School, The University of Sydney, Sydney, NSW Australia; 9 Centre for Healthcare Transformation, School of Nursing, Faculty of Health, Queensland University of Technology, Brisbane, Australia; 10 Oncology, Concord Repatriation General Hospital, Concord, NSW Australia; 11 Palliative Care, South West Sydney Local Health District, Bankstown, NSW Australia; 12 Sacred Heart Supportive & Palliative Care, St Vincent’s Hospital Sydney, Darlinghurst, NSW Australia; 13 Pharmacy, Concord Repatriation General Hospital, Concord, NSW Australia; 14 Consumer Representative, Improving Palliative, Aged and Chronic Care Through Clinical Research and Translation (IMPACCT), Faculty of Health, University of Technology Sydney, Sydney, NSW Australia; 15 Flinders Ageing Alliance, Flinders University, Bedford Park, South Australia Australia; 16 Cardiovascular Medicine, Mayo Clinic, Scottsdale, AZ USA; 17 School of Nursing, College of Health and Medicine, University of Tasmania, Sydney, NSW Australia; 18 Palliative Care, Northern NSW Local Health District, Lismore, NSW Australia; 19 Research Centre for Palliative Care, Death and Dying, Flinders University, Adelaide, South Australia Australia; 20 Territory Palliative Care – Central Australia, Alice Springs, Northern Territory Australia

**Keywords:** Neuropathic, Cancer, Pain, Trials, Experience, Palliative, Hope, Purpose, Impact, Recruitment

## Abstract

**Background:**

Optimal intervention and clinical trial designs require consumer contributions. The experience of people with advanced cancer and palliative care needs in neuropathic pain trials is seldom reported.

**Methods:**

This exploratory descriptive qualitative sub-study aimed to understand the experience of patients and carers participating in the Lidocaine for Neuropathic Cancer Pain (LiCPain) study, a pilot randomised controlled trial of continuous subcutaneous infusion of lidocaine hydrochloride or placebo over 72 h in people with unrelieved neuropathic cancer pain. All participants enrolled in the trial, conducted at five Australian palliative care inpatient sites, were intended to be invited to participate at the time of consenting to the main study. Reasons for not being invited, consenting to or completing the interview sub-study were not collected. A single face-to-face or telephone interview was audio recorded by the site study team nurses or doctors using a semi-structured interview guide. Data were analysed following Braun and Clarke thematic analysis approach.

**Results:**

Out of 17 participants randomised to the LiCPain trial, seven participants and one carer consented to participate in the qualitative sub-study.

Three major themes were identified:

• Trial participation offered a sense of hope and purpose;

• The impact of the intervention has multiple contributing factors; and

• Pain impacts every aspect of life.

**Conclusions:**

Participants found hope and purpose in the Lidocaine for Neuropathic Cancer Pain trial. Contextual factors influenced perceived effectiveness. These findings will inform future intervention designs and clinical trials to improve outcomes for people experiencing unrelieved neuropathic cancer pain.

**Trial registration:**

This trial was registered in the Australian New Zealand Clinical Trials Registry (ACTRN12617000747325) on 22nd May 2017.

**Supplementary Information:**

The online version contains supplementary material available at 10.1186/s12904-026-02043-x.

## Background

Optimal clinical trial design and governance requires consumer contribution. Understanding participants’ experiences in feasibility studies allows trial and intervention designs to be adapted to ensure that trials are feasible and solutions meet the needs of the target population. People with advanced cancer may have competing priorities and low capacity to engage in research design, so qualitative interviews allow the voices of this group of people to be heard.

Few extant studies of interventions for neuropathic pain in advanced cancer include a qualitative component; most collected data from cancer survivors or people with good performance status [1–4]. It is important to understand the experience of people with more advanced cancer living with neuropathic pain.

We conducted a pilot randomised controlled trial of continuous subcutaneous infusion of lidocaine hydrochloride 10% w/v (3000 mg/30 mL) or placebo (sodium chloride 0.9%) over 72 h in people with unrelieved neuropathic cancer pain. This study found that the completion rate of study medication and procedures was 93% (95% confidence interval 88%−98%) and that 88% of participants completed 72 h of study medication. Treatment-emergent adverse events were infrequent and generally mild or moderate nervous system, cardiac and vascular abnormalities. Both intervention and control groups demonstrated a reduction in pain intensity with no significant difference. This qualitative study was embedded within the published pilot randomised controlled trial [5, 6] (registration: ACTRN12617000747325, 22nd May 2017) [7].

## Methods

### Aim

To understand the experience of patients and carers participating in the LiCPain randomised controlled trial and the acceptability and feasibility of the proposed trial design.

### Study design

An exploratory descriptive qualitative [8] sub-study of participants and their carers in the LiCPain pilot randomised controlled trial, using semi-structured interviews.

### Setting

This study was conducted across five Australian palliative care inpatient settings with between 17 and 28 beds and 350 to 670 admissions per year, during 2019–24.

### Participant selection and method of approach

All participants in the pilot double-blind randomised controlled parallel-group pilot and their carers were intended to be invited by the study nurse or investigator to participate in a qualitative interview at the time of consenting to the main study. Reasons for not being invited, consenting to or completing the interview sub-study were not collected.

Thematic sufficiency was assessed during analysis, and it was felt that the data was of adequate richness to allow generation of meaningful themes [9]. Further diversity of perspectives, particularly carer perspective would have enhanced this data, however sample size was limited by the pilot randomised controlled trial recruitment pool.

### Data collection

A single face-to-face or telephone interview was conducted with each consenting participant within two days of completion of the primary endpoint (day four to six) using a piloted semi-structured interview guide (Table 1 and Additional File 1). Face-to-face interviews were conducted alone at the participant’s bedside or in a quiet space on the ward. The interview was audio-recorded and transcribed verbatim by DeliverHealth, Inc and confirmed by JL. Field notes were not taken. Transcripts were deidentified and were not linked to the trial arm. Transcripts and findings were not returned to participants for checking due to the length of time between interview and transcription and the poor prognosis of the participants. Demographic and clinical data was extracted from the main LiCPain trial database with preservation of blinding.

**Table 1:** Patient interview guide

Topic	Initial open questions	Possible probing questions
Overall study	How have you found being involved in this study?	What things did or didn’t you like about being involved? Are there any changes you would recommend? Would you recommend this study to another patient? Why/Why not?
Feasibility of phase III	What concerns did you have about participating in this study?	Were there any things that may have stopped you from participating initially? What positives and negatives did you find from participating in the study? Did you worry about being in the placebo arm?
Specific components (if not already covered)		Using a syringe driver Subcutaneous route Hospitalisation Assessments Having other medications unchanged during the study
Translation to practice	Would you use this treatment if it was found to be effective?	What would make you more or less likely to try this treatment outside of a trial?
Pain experience	How has having pain affected you?	Are there any aspects of how pain affects you that we haven’t assessed in this trial which you would like to discuss?

### Research team and reflexivity

The research team comprised female and male Australian clinicians, academic researchers and a consumer. Interviewers were female registered nurses and a palliative care physician (JL) from the study team at each site, often with established relationships with participants from the trial or clinical care. Participants were aware of the purpose of the study as discussed in the consent process, but not the researchers’ personal motivations. During the consent process it was emphasised that the trial and interview was voluntary and would not impact clinical care, in order to reduce the risk of coercion, and where possible a researcher separate to the clinical team was involved. The principal investigator was a female PhD candidate and admitting physician at the lead site with prior practical and theoretical experience in qualitative methodology, guided by experienced team members. Reflexivity was practiced during design, data collection and analysis through reflection, peer debriefing and collaboration. Pre-existing biases were considered during data analysis through reflection and the inclusion of a researcher (EH) external to the original randomised controlled trial team.

### Data analysis

Data were analysed following Braun and Clarke’s reflexive thematic analysis approach [10, 11]. Thematic analysis [10, 11] was grounded in a pragmatist research paradigm [12]. Six steps were followed [13].

- *Familiarisation with the dataset*. Interviews were transcribed by a professional transcription service. No field notes were taken. The interviews were reviewed (JL), and transcripts confirmed (JL) and deidentified (JL).
- *Coding*. All transcripts were independently coded by two researchers (JL and EH), with some transcripts having a third coder (JLP or ML). Disagreements were handled through discussion between coders. Data were manually coded into categories without fitting into a pre-existing coding frame or researcher preconception. Codes generated by the researcher were formatted into tables with relevant quotes using Microsoft Word. The coding tree is available in Additional file 2.
- *Generating initial themes*. Codes were combined with other codes to form potential overarching themes and sub-themes (JL, EH) and refined (JL, JLP, ML, MA).
- *Developing and reviewing themes*. The themes were reviewed in relation to the coded extracts and the entire dataset (JL, JLP, ML, MA). Interpretive differences were resolved through discussion.
- *Refining, defining and naming themes*. Themes were further refined and named (full investigator team).
- *Writing up*. The initial draft report was written by JL and refined by all investigators.

### Ethical considerations and reporting

This study was approved by the Sydney Local Health District HREC 2019/ETH07984. Written consent was obtained from each participant. The voluntary nature of participation was emphasised, including participants’ freedom to withdraw at any time. No incentives were offered. Reporting of this study adhered to the COREQ checklist [14].

## Results

### Demographics

**Fig. 1:**
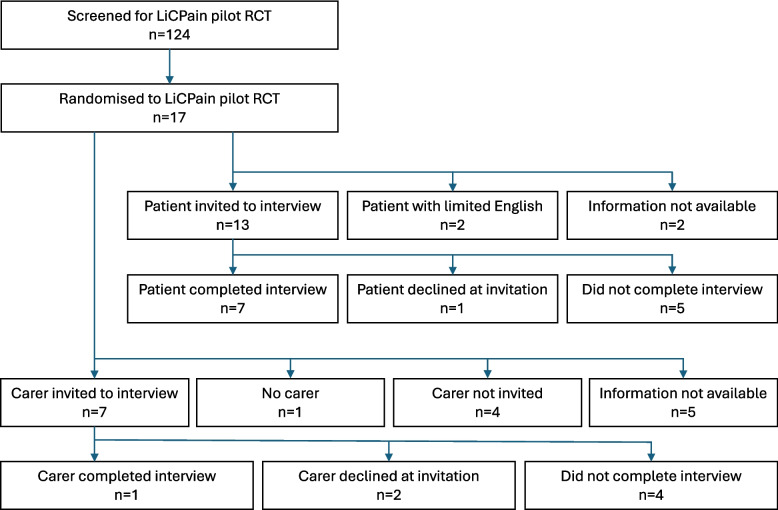
Participant flow diagram

Seven of the 17 participants randomised to the LiCPain trial (P1–7) consented and contributed to the qualitative sub-study. One of the seven interviewed patients had a participating carer (P1) (Fig. 1). The semi-structured interviews ranged in length from 15 to 60 min. As only one carer was interviewed, these data were integrated with the patient themes rather than analysed as a separate dataset.

Interviewed patients were between 41 and 75 years old (median 70 years), and five were female. Their primary cancers were breast, colorectal, prostate or urological. All participants usually spoke English at home. The median worst pain score was 8 on a 10-point numeric rating scale (range 7–10) and the baseline oral morphine equivalent intake was 30 to 285 mg daily. Their median baseline Australia-modified Karnofsky Performance Status [15], which measures performance status on a scale from zero (dead) to 100 (normal, no complaints, no evidence of disease), was 40 (range 20–60).

### Main findings

Three major themes were identified:

1. trial participation offered a sense of hope and purpose;
2. the impact of the intervention has multiple contributing factors; and
3. pain affects every aspect of life.

### Theme 1: trial participation offered a sense of hope and purpose

#### Sub-theme 1A: altruism and legacy

Many participants (5/7 patients) identified their rationale for being in the trial as to help other people. Participant P2 wanted to be in the trial:

*Just for the sake of helping out.*

Participant P3 explained their philosophy that:

…participating in research is for the good of all.

Study participation met their desire to continue learning as an individual and as a society.

*I have a real thing about learning and what you need to do. And you know, that if I want to benefit from any of these sort of research things, then I need to also be a part of doing it ... If we don’t actually participate, then we’re not going to get any benefit from any of the changes; nothing is going to change. And in fifty years time, it'll still be exactly the same.* (P4)

Participants felt proud that they would leave a meaningful legacy. Participant P5 stated:

*It’s the future of medicine, you know. I mean it’s us, but there’s a lot of people that might be helped by it.*

#### Sub-theme 1B: trial participation: a help or hindrance?

When deciding whether to take part in this trial, participants weighed the risks and benefits for themselves. Key concerns were the risk of side effects and the inconvenience of participating in the trial.

*At the back of my mind, I was just thinking of the potential side effects. Because I am quite sensitive to medication, I didn’t know how I would react.* (P3)

Participants balanced this concern with the hope that the trial would broaden their therapeutic options and relieve their pain, as well as provide the satisfaction of helping others. A carer said the following about her husband:

*… when he was on the trial, I think it was a positive sign for him. He probably thought “This is it. This is something that's going to take away this dreaded pain.”* (C1)

#### Sub-theme 1C: monitoring and control can assuage uncertainty

Uncertainty about their clinical condition before, during and after the trial distressed some participants:

*What is affecting me is driving here every morning thinking “what am I going to find?” Once I get here and see what's happening, then I just get on with it.* (C1)

Some participants (3/7 patients and C1) reported plans for close monitoring eased concerns about uncertainty and adverse effects. Participant P3 stated:

*I was well monitored by [researcher]. So I was at ease after, like, a few hours of participating kind of knowing that I was monitored. If something happened, I was kind of in good hands.*

The importance of maintaining control was mentioned in different settings. Participant P5 valued being in control of trial participation:

*… so long as it wasn’t going to increase the pain at all, and I knew that any time if it did, I could stop.*

Some participants (3/7 patient and C1) valued verbal and written communication about management plans. The carer described how a written plan on a whiteboard in the room contributed to a sense of control.

*[it was helpful] to know what drugs were coming, what drugs were to be given at certain times, what we could do to ease.* (C1)

#### Sub-theme 1D: acceptable despite frustrations

Most participants (6/7 patients and C1) felt trial processes were “very easy” (P4) and overall had a positive experience.

*I didn't have to involve myself too much, I just had to be the subject of examination. So I didn't feel that there was much work required from me other than providing any medical things I was feeling at the time.* (P1)

Some participants (2/7) found the assessments “a little bit repetitive” (P2) and tiring:

*It nearly wore me out.* (P5)

Participants did not report worry about changes to usual management on the trial such as keeping medications unchanged.

Staff professionalism, organisation, flexibility and consideration for participants were valued:

*I’m very thankful that the research staff were very flexible and would say, “come back here at two o’clock” or “go and take a break.”* (P3)

Participant P6 found change in trial staff difficult:

*I find that my only difficulty with a team approach is that I’m talking to a different person each time.*

All participants stated they would recommend study participation to others in the future.

*I definitely recommend the study for other people.* (P3)

### Theme 2: the impact of the intervention has multiple contributing factors

#### Sub-theme 2A: contextual factors

The perceived effect of the intervention had multiple dimensions that were unrelated to its pharmacological action, including hope and preconceptions.

Being on the trial affected the participants’ mental state, which in turn affected their pain and function. Carer C1 noted her partner’s pain was interrelated with his mood.

*… it just seems the pain comes and goes ... it all depends [on] his mood.*

C1 believed the trial reduced her partner’s pain and improved his functional capacity.

*On the trial he was a lot more upbeat, can do things. And when the trial ended on Saturday, on Sunday he was just down and [feeling] pain and not able to do things. He couldn't feed himself, nothing. So maybe that was his way of thinking, oh well, something else is being taken away.*

However, she felt that it was hard to determine the cause of his changes in pain because:

*… every day is different with his pain.* (C1)

One participant identified that their prior experience may have influenced how they felt. Participant P6 noted they were prone to getting side effects from medications:

*My side effects were just a general giddiness that I tend to get with a lot of medications.*

And that this experience may have contributed to the physical impact of the medication.

*… unfortunately from almost sort of on the first day, the effects I felt caused my debilitation in the way that I suddenly – whether it was psychosomatic or not, I don’t know – but I suddenly felt there were so many things I couldn’t do without assistance. I couldn’t get to the toilet, and prior to the trial, I could quite easily get to the toilet.* (P6)

#### Sub-theme 2B: intervention delivery

Aspects of the intervention delivery affected the likelihood of a participant wanting to use this intervention in future.

Some participants (4/7) found the subcutaneous butterfly and syringe driver "not a problem at all” (P2). P4 said:

*With the little dilly bag that it was in, it made it very easy. I could get up and go and sit outside in the sun, go for walks down the end of the ward.*

While others (3/7) found it awkward, saying:

*… my shakiness and imbalance made me feel quite awkward at times with that, that I was going to drop the unit. I didn’t feel confident with that. But as regards to the actual implants themselves, I soon forgot they were there.* (P6)

A suggestion was to put the equipment in:

*… a lighter handbag. Or something, you know, more easily detachable.* (P5)

#### Sub-theme 2C: hospitalisation: a virtue or a vice?

Participants had mixed opinions about being hospitalised for the intervention. Frustrations about being in hospital included logistical problems (such as parking) as well as a lack of control.

*I’m used to being at home on my own and sort of my day is nice and calm and quiet. And being interrupted all the time by different people coming in for different reasons – but that’s hospital ... I want to sleep in my bed, and I want to get my drugs as soon as I want them, not to have that long wait.* (P4)

Other participants saw the benefits of being in the hospital as:

*If you're in a safe environment, given your condition, et cetera, it's probably better to be in the hospital.* (P1)

#### Sub-theme 2D: embrace the intervention if effective

Overall, most participants (6/7) stated that they would use this medication if it was found to be effective.

*I’ll just definitely give it a go.* (P2)

Some participants (3/7) qualified this statement, stating they would need adjustments to dose or delivery, or:

*… would like to see some sort of results from somewhere that says, you know, like there’s a seventy per cent chance that this will work for you.* (P4)

### Theme 3: pain impacts every aspect of life

#### Sub-theme 3A: pain impacts daily life

Pain impairs function not only by restricting movement, but by causing fatigue and hindering communication. This was expressed through sentiments such as:

*… [pain] stop[s] you doing the stuff you want to do.* (P7)

*It’s made me exhausted ... so tired you can’t read.* (P5)

*I find it difficult to communicate when I’m in pain.* (P6).

Pain was repeatedly described as affecting a person’s whole life, with participants giving descriptions such as:

*It steals my, I suppose, general wellbeing.* (P6)

*It affects my everyday life*. (P3)

The impact of the limitation from neuropathic cancer pain was described as:

*… overwhelming at times.* (P6)

#### Sub-theme 3B: impact on relationships

Some participants’ (2/7 patients and C1) found pain affected their relationships with friends and family by hampering their communication and ability to participate in activities. Participant P4 described how her experience was not understood:

*You look at your friends and family, and there are some of them who understand and there are some of them who obviously don’t. And who get very resentful for the fact that, you know, you’re not able to do things; you’re not able to keep up.*

In particular, this participant felt that others did not understand the changing nature and uncertainty of neuropathic cancer pain.

*Now I’ll say, “Look, I’ll try,” and that gets people’s back up. It’s, you know, “but you went to [friend’s event] and you’re not coming to mine.” Yeah well, I didn’t have pain that day.* (P4)

#### Sub-theme 3C: grief and loss

Participants spoke of the grief they felt when comparing their current situation to their previous condition. Participant P4 reflected how she was very healthy previously:

*Until I got cancer, I’d never even had a headache.* (P4)

She described her grief at the loss of her rich former life.

*I can’t be involved in a lot of the things that I used to be involved in. I’ve had to change the way I do things. It’s made me change my whole life, my living, my working.* (P4)

C1 also spoke of how her partner’s pain affected her own quality of life.

*It's very tough on somebody who is always on the go, and now it's just hospital and home and hospital room. I would like to take him there [to a café] to sit down and have lunch, and I can't do that ... Well we can't do any of that now.*

## Discussion

### Main findings in relation to existing evidence

Our qualitative sub-study findings indicate that participation in the LiCPain trial provided a sense of hope and purpose for interviewed patients, revealing important insights into the complex nature of neuropathic cancer pain and its management. This finding aligns with a growing body of research identifying hope and altruism as key drivers in anti-cancer therapy clinical trials [16–19], showing that these factors are also relevant in cancer pain trials. Our findings support other findings of the significant impact of cancer pain on every element of quality of life, including roles, relationships, wellbeing and even meaning and purpose [20–22].

Patients in this study found meaning and purpose in trial participation because it enabled them to create a legacy. Despite altruistic motivations, patients carefully evaluated the personal benefits of trial participation against the risks and burden. This is consistent with previous work finding patients’ perceptions of anticipated burden affected their decision to participate [4]. In this study, altruism was a prominent theme, whereas in early-phase studies it is seen as a secondary driver of trial participation [19]. Altruistic motives may have contributed to a high completion rate in the LiCPain trial [5], including for participants who ceased the infusion early. Participants expressed the importance of hope, including to be able to help future patients, leave a legacy and improve pain, concepts parallel to those of participants in early-phase studies who hope for a cure [17, 18] or to extend life [19]. Research on participating in palliative care trials is predominantly confined to the evaluation of hypothetical participation in research, with few studies describing the experience of actual trial participation [23–25]. The extant studies identify similar themes of benefits for self and others. These findings demonstrate nuanced decision-making processes and highlight that clearly communicating risk management strategies in trial recruitment with patients living with advanced cancer can increase their sense of control, which is important for patients.

Contextual factors [26] contributing to a placebo response [27] are less well studied in cancer pain than chronic pain. Nonetheless, the impact of all components of the intervention – including the delivery, therapeutic alliance and patient factors, as well as the pharmacology of the medication – must be considered in both clinical practice and trial design [28, 29]. Our data suggests contextual factors such as participant beliefs about the benefit of the trial or sensitivity to medication may influence the positive and adverse effects of an intervention. These perspectives may have contributed to the reduction of pain intensity in both arms of the LiCPain trial [5].

Although the experiences described were specific to the LiCPain intervention, some aspects of delivery utilised in operationalising this trial are also used to provide clinical management in palliative care, such as hospitalisation or use of a subcutaneous syringe driver. Syringe driver redesign to reduce bulk and improve useability may improve acceptability, which is consistent with previous reports [30]. The participants’ mixed perspectives on hospitalisation highlight the need for choice in delivery of care, which links back to the sub-theme of control.

Our findings emphasise the devastating impact of neuropathic cancer pain on multiple life domains in this group, consistent with studies in cancer pain [31], bone pain [32] and chemotherapy-induced peripheral neuropathy [33, 34]. Participants experienced grief at the loss of what used to be “normal” before living with neuropathic cancer pain, which has been reported in people with chemotherapy-induced cancer pain [35]. Few previous studies specifically examined the experience of neuropathic cancer pain, which is known to worsen physical, cognitive and social function more than nociceptive pain [36].

### Strengths and limitations of the study

This qualitative sub-study provides valuable insights into the experience and thought processes of people with advanced cancer who were interviewed as part of a neuropathic pain intervention trial. It gives voice to a vulnerable population often excluded from research due to their frailty, offering rare insights into their trial participation motives, intervention acceptability, and the profound impact of neuropathic cancer pain on daily life and relationships.

This data captures only a sample of potential participants in this trial, creating bias by capturing the views of those who chose to participate in the trial and again of those who chose or were able to participate in an interview. Understanding the reasons for not participating or being invited would have provided better context for the findings. Cultural diversity was limited by the requirement to speak English. This may not fully reflect the true barriers contributing to recruitment difficulties or the experience of all people with neuropathic cancer pain. The single carer interview limits the transferability of the carer perspective and should be interpreted cautiously.

The dual role of some interviewers as both researchers and treating physicians may have influenced the patients’ and carer responses. This may lead to significant social desirability bias [37], particularly regarding the acceptability of the intervention and motivation for undertaking the trial, due to a desire to please the clinical team or appear in a positive light. Interpretations could not be verified with participants. These limitations should be considered when interpreting the results. However, given the paucity of data on people with advanced cancer undergoing neuropathic pain trials in palliative care, our results make a useful contribution to knowledge about their experiences and their likeness to those of similar populations.

### Implications for research and clinical practice

Clinicians need to be cognisant of the value of trial participation and the self-efficacy it affords for many patients, as well as the importance of allowing them to determine whether to participate. Empowering patients to make this decision if they are eligible will improve recruitment [38]. The findings of this study may also help to improve future neuropathic cancer pain trial designs by highlighting the potential benefit of the trial to others in patient information and consent forms and other recruitment materials [38], or in a certificate of appreciation [39]. Resources are available to assist in embedding this process in clinical design [40]. Future trial designs could improve intervention acceptability by offering lidocaine infusion out of hospital with sufficient supports to ensure participant safety. Finally, patients valued the analgesic benefit of the intervention and noted its positive impact on their psychological state.

## Conclusions

This study found that interviewed participants in the LiCPain trial who were living with advanced cancer and neuropathic cancer pain derived hope and purpose from their involvement. Contextual factors may have influenced the intervention’s perceived effectiveness, emphasising the need for patient-centred trial and intervention design. Participants valued having a sense of control, which can be enhanced by reducing uncertainty and minimising burden. Findings from this study can be used to refine the design of future clinical trials of interventions for neuropathic cancer pain. The substantial burden of pain for participants highlights the urgent need for clinical trials of ways to improve outcomes for people suffering from unrelieved neuropathic cancer pain.

## Supplementary Information

Additional file 1: Career interview guide

Additional file 2: Coding tree

Additional file 3: Reporting checklist

## Data Availability

The datasets and coding tables analysed during the current study are available from the corresponding author on reasonable request.
