# Supplementary material for: Feasibility and acceptability of “LiCPain” pilot randomised controlled trial of continuous subcutaneous infusion of lidocaine or placebo for people with neuropathic cancer pain: a qualitative study of patient and carer perceptions and experiences
Source: BMC Palliat Care. 2026 Mar 17;25:111. doi: 10.1186/s12904-026-02043-x (PMC13107867; doi:10.1186/s12904-026-02043-x)
Supplement: Supplementary file 1 — Additional file 1: Career interview guide [file 12904_2026_2043_MOESM1_ESM.pdf]

Additional File 1: Carer Interview Guide

Topic	Initial open questions	Possible probing questions
Overall study	How have you found the experience of (name) being involved in this study?	What things did or didn't you like about (name) being involved?<br>Are there any changes you would recommend?<br>Would you recommend this study to another patient? Why/Why not?
Feasibility of phase III	What concerns did you have about (name) participating in this study?	Were there any things that may have stopped (name) from participating initially?<br>What positives or negatives did you find from (name) participating in the study?<br>Did you worry about (name) being in the placebo arm?
Specific components (if not already covered)		Using a syringe driver<br>Subcutaneous route<br>Hospitalisation<br>Assessments<br>Having other medications unchanged during the study
Translation to practice	Would you recommend this treatment if it was found to be effective?	What would make you more or less likely to recommend this treatment outside of a trial?
Carer experience	How has (name) having pain affected you?	Are there any aspects of how pain affects (name) that we haven't assessed in this trial which you would like to discuss?
