# Supplementary material for: Feasibility and acceptability of “LiCPain” pilot randomised controlled trial of continuous subcutaneous infusion of lidocaine or placebo for people with neuropathic cancer pain: a qualitative study of patient and carer perceptions and experiences
Source: BMC Palliat Care. 2026 Mar 17;25:111. doi: 10.1186/s12904-026-02043-x (PMC13107867; doi:10.1186/s12904-026-02043-x)
Supplement: Supplementary file 2 — Additional file 2: Coding tree [file 12904_2026_2043_MOESM2_ESM.pdf]

Additional File 2: Coding Tree

Theme	Sub-theme	Codes
Trial participation offered a sense of hope and purpose	Altruism and legacy	• Reasons to recommend (future patients)<br>• Value of participation
	Trial participation: a help or hindrance?	• Ease of participation<br>• Concerns about participating (monitoring AE, potential AE)
	Monitoring and control can assuage uncertainty	• Monitoring of adverse effects (reassured by surveillance)<br>• Uncertainty<br>• Long-term management of pain
	Accceptable despite frustrations	• Experience with the study (positive/negative/neutral)<br>• Attitude towards placebo<br>• Assessments and questionnaires (positive feedback, flexible scheduling)<br>• Recommend study
The impact of the intervention has multiple contributing factors	Contextual effects	• Attitude towards placebo<br>• Adverse effects of use (e.g. drowsiness)<br>• Effectiveness of intervention<br>• Multifactorial pain management
	Intervention delivery	• CSCI device use (convenience, size, SC administration, walking with SD)
	Hospitalisation: a virtue or a vice?	• Hospital experience (inpatient stay)
	Embrace the intervention if effective	• Future considerations<br>• Would use if effective<br>• Attitude towards opioids
Pain impacts every aspect of life	Pain impacts daily life	• Impact on daily life<br>• Description of pain
	Impact on relationships	• Pain not understood by friends or family
	Grief and loss	• Change in pain<br>• Comparing self to others
