# Supplementary material for: Feasibility and acceptability of “LiCPain” pilot randomised controlled trial of continuous subcutaneous infusion of lidocaine or placebo for people with neuropathic cancer pain: a qualitative study of patient and carer perceptions and experiences
Source: BMC Palliat Care. 2026 Mar 17;25:111. doi: 10.1186/s12904-026-02043-x (PMC13107867; doi:10.1186/s12904-026-02043-x)
Supplement: Supplementary file 3 — Additional file 3: Reporting checklist [file 12904_2026_2043_MOESM3_ESM.pdf]

Consolidated criteria for reporting qualitative studies (COREQ): 32-item checklist

No	Item	Guide questions/description	Section	Excerpt
**Domain 1: Research team and reflexivity**				
Personal Characteristics				
1.	Interviewer/facilitator	Which author/s conducted the interview or focus group?	Methods – Research team and reflexivity, paragraph 1	“Interviewers were female registered nurses and a palliative care physician (JL) from the study team at each site…”
2.	Credentials	What were the researcher's credentials? *E.g. PhD, MD*	Author list	“Jessica T. Lee MBBS, FRACP, FAChPM…”
3.	Occupation	What was their occupation at the time of the study?	Methods – Research team and reflexivity, paragraph 1	“Australian clinicians, academic researchers and a consumer.”
4.	Gender	Was the researcher male or female?	Methods – Research team and reflexivity, paragraph 1	“The research team comprised female and male Australian clinicians…”
5.	Experience and training	What experience or training did the researcher have?	Methods – Research team and reflexivity, paragraph 1	“The principal investigator was a female PhD candidate…with prior practical and theoretical experience in qualitative methodology…”
Relationship with participants				
6.	Relationship established	Was a relationship established prior to study commencement?	Methods – Research team and reflexivity, paragraph 1	“…often with established relationships with participants from the trial or clinical care.”
7.	Participant knowledge of the interviewer	What did the participants know about the researcher? e.g. *personal goals, reasons for doing the research*	Methods – Research team and reflexivity, paragraph 1	“Participants were aware of the purpose of the study as discussed in the consent process, but not the researchers’ personal motivations.”
8.	Interviewer characteristics	What characteristics were reported about the interviewer/facilitator? e.g. *Bias, assumptions, reasons and interests in the research topic*	Methods – Research team and reflexivity, paragraph 1	“Interviewers were female registered nurses and a palliative care physician (JL) from the study team at each site…”
**Domain 2: study design**				
Theoretical framework				
9.	Methodological orientation and Theory	What methodological orientation was stated to underpin the study? *e.g. grounded theory, discourse analysis, ethnography, phenomenology, content analysis*	Methods – Data analysis, paragraph 1	“Data were analysed following Braun and Clarke’s reflexive thematic analysis approach.”

Participant selection				
10.	Sampling	How were participants selected? *e.g. purposive, convenience, consecutive, snowball*	Methods – Participant selection, paragraph 1	"All participants in the pilot double-blind randomised controlled parallel-group pilot and their carers were intended to be invited…"
11.	Method of approach	How were participants approached? e.*g. face-to-face, telephone, mail, email*	Methods – Participant selection, paragraph 1	"…by the study nurse or investigator to participate in a qualitative interview at the time of consenting to the main study."
12.	Sample size	How many participants were in the study?	Results – Demographics, paragraph 1	"Seven of the 17 participants … consented and contributed to the qualitative sub-study. One of the seven interviewed patients had a participating carer."
13.	Non-participation	How many people refused to participate or dropped out? Reasons?	Figure 1	Figure 1
Setting				
14.	Setting of data collection	Where were the data collected? e.*g. home, clinic, workplace*	Methods – Data collection, paragraph 1	"A single face-to-face or telephone interview was conducted … Face-to-face interviews were conducted alone at the participant's bedside or in a quiet space on the ward."
15.	Presence of non-participants	Was anyone else present besides the participants and researchers?	Methods – Data collection, paragraph 1	"Face-to-face interviews were conducted alone at the participant's bedside."
16.	Description of sample	What are the important characteristics of the sample? *e.g. demographic data, date*	Results – Demographics, paragraph 2	"Interviewed patients were between 41 and 75 years old…"
Data collection				
17.	Interview guide	Were questions, prompts, guides provided by the authors? Was it pilot tested?	Methods – Data collection, paragraph 1	"…using a piloted semi-structured interview guide (Table 1 and Additional File 1)."
18.	Repeat interviews	Were repeat interviews carried out? If yes, how many?	Methods – Data collection, paragraph 1	"A single face-to-face or telephone interview was conducted with each participant."
19.	Audio/visual recording	Did the research use audio or visual recording to collect the data?	Methods – Data collection, paragraph 1	"The interview was audio-recorded and transcribed verbatim …"
20.	Field notes	Were field notes made during and/or after the interview or focus group?	Methods – Data collection, paragraph 1	"Field notes were not taken."
21.	Duration	What was the duration of the interviews or focus group?	Results – Demographics, paragraph 3	"The semi-structured interviews ranged in length from 15 to 60 minutes."
22.	Data saturation	Was data saturation discussed?	Methods – Participant selection, paragraph 2	"Thematic sufficiency was assessed during analysis…"

23.	Transcripts returned	Were transcripts returned to participants for comment and/or correction?	Methods – Data collection, paragraph 2	"Transcripts and findings were not returned to participants due to the poor prognosis of the participants."
**Domain 3: analysis and findings**				
Data analysis				
24.	Number of data coders	How many data coders coded the data?	Methods – Data analysis, paragraph 1	"Two researchers (JL and EH) initially coded the transcripts, with some transcripts having a third coder."
25.	Description of the coding tree	Did authors provide a description of the coding tree?	Additional file 2	Additional file 2
26.	Derivation of themes	Were themes identified in advance or derived from the data?	Methods – Data analysis, paragraph 1	"Codes were combined with other codes to form potential overarching themes and sub-themes."
27.	Software	What software, if applicable, was used to manage the data?	Methods – Data analysis, paragraph 1	"Codes generated by the researcher were formatted into tables with relevant quotes using Microsoft Word."
28.	Participant checking	Did participants provide feedback on the findings?	Methods – Data collection, paragraph 1	"Transcripts and findings were not returned to participants for checking due to the length of time between interview and transcription and the poor prognosis of the participants."
Reporting				
29.	Quotations presented	Were participant quotations presented to illustrate the themes / findings? Was each quotation identified? e.g. *participant number*	Results – Main findings	Quotations presented and identified with P1-7 and C1.
30.	Data and findings consistent	Was there consistency between the data presented and the findings?	Results – Main findings	Themes and subthemes were carefully derived from the data.
31.	Clarity of major themes	Were major themes clearly presented in the findings?	Results – Main findings, paragraphs under each theme	Three major themes were identified:<br>1) trial participation offered a sense of hope and purpose;<br>2) the impact of the intervention has multiple contributing factors; and<br>3) pain affects every aspect of life.
32.	Clarity of minor themes	Is there a description of diverse cases or discussion of minor themes?	Results – Main findings	Minor themes were discussed within sub-themes. Diverse cases were reported where appropriate eg. "Some participants found the subcutaneous butterfly and syringe driver "not a problem at all" (P2)...While others found it awkward"
